# Ready-to-use or Powder/Liquid Bioceramic Sealer: Micro-CT analysis of root canal filling removal according to ultrasonic agitation

**DOI:** 10.4317/jced.62728

**Published:** 2025-10-01

**Authors:** Marcia Eugênia de S- D Feitosa, Ana Grasiela Limoeiro, Raimundo S. de Oliveira-Neto, Suyane Maria Luna-Cruz, Rodrigo Ricci Vivan, Murilo Priori Alcalde, Marco Antônio Húngaro Duarte, Bruno Carvalho de Vasconcelos

**Affiliations:** 1 Post-graduate Program in Dentistry, Faculty of Pharmacy, Dentistry and Nursing, Federal University of Ceará, Fortaleza, CE, Brazil; 2 Department of Dentistry, Endodontics and Dental Materials, Bauru Dental School, University of São Paulo, Bauru, SP, Brazil

## Abstract

**Background:**

This study evaluated the effectiveness of ultrasonic agitation in removing residual root canal filling material (RFM) from mandibular molars with isthmuses and to assess the influence of different bioceramic endodontic sealers, Bioroot RCS (BCS), and EndoSequence BC Sealer (ESBC).

**Material and Methods:**

Forty human mandibular molars were prepared with Reciproc R25 and divided based on the obturation material. The retreatment was performed (Reciproc R40) and the roots were randomly divided between agitation protocols (PUI/CUI) using a large solution volume (60 mL/canal).

**Results:**

The percentage reduction of RFM was measured after agitation. Ultrasonic agitation resulted in a percentage reduction of RFM ranging from 54.39% to 68.10% in the total area, and from 56.25% to 73.21% in the apical third (*P* < .05). No significant differences were found related to sealer type or activation protocol (*P* > .05).

**Conclusions:**

While none of the protocols completely removed RFM, ultrasonic agitation proved effective in its reduction, independent of the sealer presentation and agitation method used.

** Key words:**Endodontics, Ultrasonic Agitation, Root Canal Irrigants, Root Canal Filling, X-ray Microtomography.

## Introduction

Endodontic retreatment aims to solve cases where the original treatment has failed [1], offering the best prognosis and a high success rate [2]. However, its execution can be challenging [3], particularly in flattened canals, lateral canals, or isthmuses, where instruments struggle to reach efficiently [4].

To date, no study has demonstrated that different retreatment protocols can completely remove root canal filling material (RFM) [5-7]. The obturation material plays a significant role in retreatment as it seals the root canal and fills irregularities [8]. While much research has focused on removing resin-based sealers during endodontic retreatment [9,10], little has been investigated regarding bioceramic sealers.

These sealers, which contain calcium silicates as the main components, were developed to offer good physicochemical properties [11] and excellent biocompatibility and bioactivity [12]. They are available in two forms: ready-to-use and requiring mixing [13]; the implications of this variation during retreatment are still unknown.

Given the difficulty in removing obturation materials in endodontic retreatment, irrigant activation has been suggested as an auxiliary method [14]. This is especially important in isthmus due to potential debris blockage, which reduces the penetrability of the irrigants [15].

Agitation can be performed through passive ultrasonic irrigation (PUI) [16], where the solution is intermittently replenished and agitated, or continuous ultrasonic irrigation (CUI), where the solution is continuously delivered alongside agitation. The literature does not definitively state which method is more efficient in removing obturation material [17], irrespective of the irrigant volume used. However, studies indicate that a longer contact time of the solution with the root canal and a greater volume of irrigant can reduce biofilm and RFM in the root canal system [18,19].

Thus, this study aims to evaluate the impact of ultrasonic agitation, both passive and continuous, on different forms of bioceramic sealer presentation (BioRoot RCS, which requires mixing, and Endosequence BC Sealer, ready-to-use) on the removal of residual filling material after mechanized retreatment in mesial canals of mandibular molars with isthmuses. The null hypotheses tested were that: (i) the type of ultrasonic agitation protocol does not affect the removal of obturation material, and (ii) there is no difference in the removal of bioceramic material based on its presentation form.

## Material and Methods

- Sample Size Calculation

The sample size was determined based on the study of Volponi *et al*. [1], which evaluated differences in RFM after applying three complementary protocols following endodontic retreatment in teeth filled with bioceramic cement. The mean and standard deviation data from the study were entered into www.openepi.com. To obtain a representative sample with 80% power and 95% confidence to test the alternative hypothesis, 8 specimens per group were required. To account for potential specimen loss, the number was increased to 10 specimens per group.

- Sample Selection 

The manuscript of this laboratory study was written according to the Preferred Reporting Items for Laboratory studies in Endodontology (PRILE) 2021 guidelines [20]. This study was approved by the Local Research Ethics Committee (Protocol #6.069.128). Forty-four extracted human mandibular molars from a pool of 80 freshly extracted teeth were standardized according to the following characteristics: apparently straight curvature (less than 5°) using Schneider [21] method, complete root formation and apical development, with lengths between 18- and 21-mm. Teeth with fused roots, previous endodontic treatment, calcifications, root fractures, or apical foramina larger than 200 μm were excluded from the sample. Initially, the mesial roots of the teeth were scanned using a micro-computed tomography (micro-CT) (SkyScan1174; SkyScan, Aartselaar, Belgium) with a voxel size of 19 μm, 50 kV, 800 mA, 0.8° rotation, and 1024 x 1304 resolution. The digital images were reconstructed using NRecon v1.6.4.8 software (Bruker-micro-CT), providing cross-sectional and axial images in BMP format. CTan v1.11.10.0 software (Bruker-micro-CT) was used for measurements, with the dental apex as the boundary for the analyzed areas. The aim was to confirm the classification of the root canal system (RCS) as type II according to Vertucci [22], and the presence of isthmuses according to Hsu & Kim [23].

- Canal Preparation and Filling

Coronal accesses were standardized using diamond burs #1014 and #3082 (KG Sorensen Ind. e Com. Ltda., Barueri, SP, Brazil) operated at high speed with continuous irrigation. Initial exploration and determination of the real length of the root canals were performed with type-K manual files #10 (Dentsply-Sirona, Ballaigues, Switzerland), introduced until their tip was visible through the apical foramen with the aid of a magnifying glass. The working length (WL) was set 1 mm short of the real canal length. In specimens where patency was not achieved, a C-Pilot #10 file (VDW GmbH, Munich, Germany) was used; if foramen patency was not found, the tooth was replaced.

To simulate clinical conditions, the foramen was sealed with a layer of gingival barrier (MaxDam, Maquira, Maringá, PR, Brazil) to prevent overflow of the irrigant solution. To avoid the gingival barrier from invading the canals, a #10 file was introduced before applying the product. With the WL determined, chemical-mechanical preparation of the specimens was performed using a Reciproc R25 instrument (#25/.08; VDW GmbH, Munich, Germany) operated by a VDW Silver electric motor (VDW GmbH) in Reciproc All mode. This instrumentation followed these steps:

• Peaking movements with light apical pressure were used to allow the file to progress passively. After instrumenting the cervical and middle regions, the instrument was removed and cleaned with sterile gauze, and canal patency was checked with a manual type K #10 file. Between each in-and-out movement (3 times), irrigation/aspiration with 5 mL (2.5 mL in each canal) of 2.5% sodium hypochlorite (NaOCl - Asfer Indústria Química, São Caetano do Sul, SP, Brazil) was carried out using an endodontic syringe and specific needles (NaviTip; Ultradent, South Jordan, UT, USA).

• Once the WL was reached, the file was removed and cleaned with sterile gauze. Instrumentation continued until no R dentin debris was visible on the file’s spirals. Patency was checked again with a manual type K #10 file. Finally, irrigation was performed with 5 mL of 17% EDTA for 3 minutes, followed by another 5 mL of 2.5% NaOCl and 10 mL of 0.9% saline solution, and patency was verified.

Each file was used only once. The canals were dried with absorbent paper points and filled with Reciproc R25 gutta-percha cones (VDW GmbH, Munich, Germany) compacted properly at the WL, 1 mm short of the apex. The filling was performed using the single-cone technique, and periapical radiographs confirmed the position of the gutta-percha cone. The teeth were randomly divided into two groups of 20 specimens each, considering the isthmus profile and canal volume in the mesial roots to ensure homogeneous distribution. The cones, when inserted, were covered with one of two bioceramic cements: BioRoot RCS (BCS) and EndoSequence BC Sealer (ESBC).

Due to differences in drying protocols, teeth assigned to BCS were aspirated for 5 seconds and dried with three absorbent paper points in each canal. Canals for ESBC were aspirated for 5 seconds and dried with only one paper point per canal. The cements were mixed/proportioned on a glass slab using a spatula, following the manufacturer’s recommendations. The cements were applied to the canals using the cone, which was then coated with cement and inserted into the canal until it reached the apical stop at the WL through vertical compaction.

Coronal excesses were removed with heated pluggers combined with cold pluggers for cervical condensation. Periapical radiographs confirmed the quality of the filling, and the access cavities were cleaned. The coronal accesses were filled with temporary material (Maxxion R; FGM, Joinville, Brazil), and the specimens had their roots immersed in a floral sponge moistened with 0.9% saline solution. Specimens were stored in an incubator (Quimis, Diadema-SP, Brazil) at 100% humidity and 37°C for 90 days.

- Canal Retreatment Procedures

After the storage period, retreatment was carried out by a single experienced operator. The temporary coronal restoration was removed using round diamond burs under abundant irrigation until the cervical portion of the filling became visible. Retreatment was performed with a Reciproc R25 file (#25/.08; VDW GmbH, Munich, Germany) at the real canal length, followed by a Reciproc R40 file (#40/.06; VDW GmbH, Munich, Germany) 1 mm short of the foramen. The instruments were operated using a VDW Silver electric motor in Reciproc All mode. Files were introduced passively until resistance was felt, then a reciprocating motion with light pressure was applied.

Once the required length was reached, filing and brushing motions were performed, particularly towards the isthmus, until no more RFM was visible on the file blades, using a stereoscopic magnifier [1]. If the instrument did not reach the WL, it was removed, cleaned with sterile gauze, and a glide path was created using a C-Pilot #10 file [24]. A second attempt was made to reach the WL. For each group, files were frequently removed and inspected for RFM and any distortions. Each file was used only once.

Throughout all steps, the irrigation protocol remained consistent with the initial instrumentation (5 mL of 2.5% NaOCl after every 3 strokes, followed by a final rinse of 5 mL 17% EDTA and 10 mL saline), without the use of solvents [25] or heating of the filling material. Periapical radiographs were taken post-retreatment to confirm the absence of filling material in the RCS. It is important to emphasize that procedures were performed sequentially, with one specimen from each group (BCS and ESBC).

- Complementary Agitation Protocols

Following the retreatment procedures, specimens were rescanned using micro-CT to evaluate the volume of RFM, using the same parameters. This allowed for the randomization of specimens between complementary irrigation protocols, considering the isthmus type and RFM volume for homogeneous distribution. Consequently, the two groups (BCS and ESBC) were further subdivided by the type of irrigant agitation: PUI or CUI, using 60 mL of 2.5% NaOCl.

Specimens underwent complementary irrigation using the Irrisonic tip (E1; Helse Ultrasonic, Santa Rosa do Viterbo, SP, Brazil) attached to the Ultrawave XS ultrasonic device (Ultradent, South Jordan, UT, USA), set at 20% power. The instrument was passively introduced until 2 mm short of the WL. Three activation cycles of 20 seconds each were conducted in each irrigation solution: the first three cycles used 20 mL of 2.5% NaOCl, while the second cycle used 2 mL of 17% EDTA for each root canal, the last cycles used 2.5% NaOCl again. the first and third cycles used 30 mL of 2.5% NaOCl, while the second cycle used 5 mL of 17% EDTA. Solutions were renewed each cycle for the PUI groups, whereas in the CUI groups, solutions were distributed continuously, with NaOCl in the ultrasonic device reservoir and EDTA delivered via syringe, totaling 60 mL of 2.5% NaOCl and 6 mL of 17% EDTA per specimen. The total activation time was 1 minute, with the insert performing short back-and-forth motions, working freely in the canal without touching the walls, and vibration directed towards the isthmus (buccal-lingual direction).

Lastly, a final irrigation with 10 mL of saline solution was administered to remove irrigation solution residues; a total of 76 mL of irrigations solutions were employed in the complementary protocols. After these complementary steps, specimens were rescanned to evaluate the volume of RFM.

- Analysis of residual filling material

The micro-CT scans were reconstructed using NRcon software (Bruker micro-CT) and aligned using the 3D registration function of DataViewer software (v.1.5.1; Bruker micro-CT). Images were processed with CTAn software (v.1.14.4; Bruker micro-CT) for quantitative analysis. For each sample, the RFM was calculated from the opening of the canal to the apical foramen, including the isthmus area and specifically within the apical third, which is 3 mm above the apical foramen. Volumes were recorded and converted to percentages to compare RFM immediately after retreatment and after supplemental treatment. The RFM volume after the supplemental irrigation protocols was determined by superimposing and comparing the images before and after these protocols.

- Statistical analysis

The RFM values before and after the additional irrigation protocols were tested for normality using the Kolmogorov-Smirnov test, which yielded non-parametric data. Therefore, the Wilcoxon signed-rank test was used to compare paired samples within each group. The percentage of RFM removal by protocols was calculated and analyzed using the paired T-test and Tukey’s post hoc test for parametric data, using a significance level of 5%.

## Results

 Table 1 shows the median, minimum and maximum values of RFM (in mm³) at two time points: before and after the final irrigation protocols, where both the entire root canal and the apical region were analyzed. Significant differences were found in all comparisons, regardless of the sealer used or ultrasonic activation (*P* < 0.05).

 Table 2 lists the mean and standard deviation of the percentage reduction in RFM achieved by each group, considering the entire tooth and only the apical region. No statistically significant differences were noted between experimental groups (*P* > 0.05).

**Figure 1:**
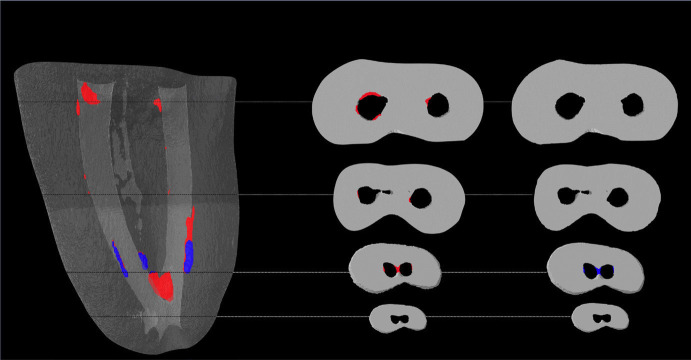
Illustrative 3D reconstruction of specimen before (red) and after (blue) ultrasonic activation protocol presenting residual filling material reduction even in root canal and isthmus area.

All samples showed RFM in the isthmus, predominantly in the middle and apical third. RFM was most frequently observed in the apical and middle thirds of the canal walls. Figure 1 shows illustrative reconstructions of RFM before and after the agitation protocols.

## Discussion

This study aimed to evaluate the influence of ultrasonic agitation on the removal of RFM after retreatment in the mesial canals of mandibular molars with isthmuses. Proper removal of these residues is crucial for enhancing the decontamination of the root canal system, as residues can shield microorganisms from the action of irrigating solutions. The study indicates that ultrasonic agitation protocols are effective in reducing RFM when using the tested bioceramic sealers. Additionally, no statistically significant differences were found between the agitation regimes, whether PUI or CUI was used, thus, the first null hypothesis was confirmed.

The mesial roots of mandibular molars were selected for this study due to their anatomical complexity, primarily the presence of isthmuses [26]. Initial scanning was crucial to ensure a homogeneous distribution of anatomical challenges across the protocols. It also ensured the standardization of chemical-mechanical preparation, obturation, and removal, all performed by a single experienced operator to reduce potential biases. Reciprocating kinematics was adopted using Reciproc instruments due to their safety, efficiency, and extensive use in the literature [5,27]. Furthermore, an exclusive analysis and enlargement of the apical region were conducted using a larger-caliber instrument (#40/.06) to address the cleaning challenges in this critical zone, known for RFM accumulation, which can lead to endodontic retreatment failure [15,25].

Regarding obturation protocols, it is important to understand that different drying sequences were employed, particularly due to the different presentations and setting kinematics. While BCS cement contains all the moisture necessary for setting in its liquid [28], ready-to-use sealers, as ESBC, uses the moisture present in the dentin for this purpose [29]. Attention to this aspect is essential for the materials to set properly, regardless of the laboratory or clinical environment.

The study’s findings corroborate previous research showing a significant reduction in RFM after using ultrasonic activation of the irrigation solution [8,30]. This reinforces the importance of employing complementary techniques in irrigation to maximize root canal filling material removal. Moreover, the volume of irrigation is closely related to the success of material removal, which is a novel aspect of this work as it utilized a 60 mL volume, considered high compared to other studies [31,32].

Regarding agitation protocol comparisons, no significant differences were observed in the percentage reduction of RFM. The literature presents mixed results, with some studies showing superiority for PUI [25] or CUI [17], while others suggest both protocols are equally effective. The statistical similarity found between the employed protocols and sealers may be due, in part, to the high volume of irrigation solution used.

Regarding the presentation form of bioceramic sealers, whether manually mixed or ready-to-use, no significant differences were observed; therefore, the second null hypothesis was accepted. This suggests that the form of presentation did not affect the presence of RFM in this study. The technical sensitivity of ready-to-use sealers concerning moisture in the RCS did not impact the difficulty of RFM removal. Currently, there is no consensus on the drying protocol for Endosequence BC Sealer, nor are manufacturers’ manuals detailed in this regard.

There is a lack of studies comparing retreatment with bioceramic sealers of different commercial presentations (i.e., ready-to-use versus powder/liquid), with comparisons between sealers of different compositions (bioceramic vs. resin-based) being more common [7], highlighting the novel aspect of this work. Additionally, this study parallels clinical routine practice, where drying protocols may not be strictly followed, affecting obturation quality, and irrigation volumes are relatively low, impairing RFM removal.

The study’s findings suggest that a high-volume irrigation protocol with ultrasonic activation effectively removes RFM after initial removal. However, the difference in removal efficacy for bioceramic sealers with different presentation forms remains unclear due to the scarcity of literature, though there appears to be no significant clinical difference when retreatment is necessary, even considering their different setting sequences.

This study has notable limitations, including the use of micro-CT analysis, which, while precise for quantifying residual filling material (RFM), may not fully replicate clinical conditions. It specifically focused on mandibular molars with isthmuses, which may not represent other tooth types. These aspects mean that the extrapolation of the results found here cannot be carried out directly to clinical practice without an effective understanding of the clinical aspects, notably possible anatomical (e.g., curved canals, multi-rooted teeth) and environmental variations, involved. Future research should investigate various bioceramic sealers and their chemical interactions with different irrigation protocols. Additionally, the effectiveness of ultrasonic activation across diverse tooth anatomies needs further exploration. Long-term outcomes of retreatment and the success of different agitation protocols should also be assessed to enhance clinical practices. Factors such as irrigant volume and type, along with the design features of sealing materials, must be analyzed to improve the applicability of the findings. Ongoing research into irrigation techniques and their impact on effective debris removal is critical for optimizing endodontic treatment outcomes.

While significant reduction in RFM was observed, complete removal remains a challenge, highlighting the ongoing need for *in vivo* studies and research across diverse tooth anatomies to fully translate these findings into improved clinical outcomes.

## Conclusions

Under the conditions of this study, it can be concluded that using an ultrasonic agitation protocol for final irrigation significantly reduces the volume of residual filling material in the mesial canals of mandibular molars. However, due to the anatomical complexity posed by the isthmus, complete removal of RFM was not achievable. Additionally, no significant differences were observed between the agitation protocols, PUI and CUI, or the form of presentation of the bioceramic sealer used.

## Figures and Tables

**Table 1:** Median, minimum and maximum, RFM values (mm3), measured before and after the use of final ultrasonic agitation protocols.

Root level	Groups	Before		After		P* value
		Median	Min.-Max.	Median	Min.-Max.	
Total	BCS /PUI	1.30	0.46 - 2.65	0.30	0.10 - 1.25	0.001
	BCS /CUI	1.32	0.96 - 2.92	0.75	0.20 - 0.90	0.001
	ESBC/PUI	0.73	0.17 - 1.76	0.12	0.00 - 0.84	0.001
	ESBC/CUI	1.64	0.10 - 2.26	0.38	0.07 - 1.36	0.001
Apical	BCS /PUI	0.39	0.14 - 0.80	0.12	0.00 - 0.57	0.0005
	BCS /CUI	0.61	0.25 - 1.12	0.24	0.02 - 0.71	0.0005
	ESBC/PUI	0.18	0.06 - 0.40	0.03	0.00 - 0.34	0.0005
	ESBC/CUI	0.36	0.03 - 1.50	0.11	0.01 - 1.26	0.0005

* Significance according to the Wilcoxon test for paired samples (*P* <.05).

**Table 2:** Table Mean values and standard deviation of RFM percentage comparing ultrasonic agitation protocol and bioceramic sealer.

Root level	Groups	Mean	SD
Total	BCS /PUI	67.46a	20.17
	BCS /CUI	54.39a	19.93
	ESBC/PUI	68.10a	27.23
	ESBC/CUI	57.92a	20.15
Apical	BCS /PUI	57.51a	24.65
	BCS /CUI	57.26a	27.58
	ESBC/PUI	73.21a	31.47
	ESBC/CUI	56.25a	18.82

a.b Different superscript letters indicate statistical significance according to the Tukey (*P* <.05).

## Data Availability

The datasets used and/or analyzed during the current study are available from the corresponding author.
